# CD40 is Positively Correlated with the Expression of Nucleophosmin in Cisplatin-Resistant Bladder Cancer

**DOI:** 10.1155/2020/3676751

**Published:** 2020-04-28

**Authors:** Chenshuo Luo, Ting Lei, Man Zhao, Qian Meng, Man Zhang

**Affiliations:** ^1^Clinical Laboratory Medicine, Peking University Ninth School of Clinical Medicine, Beijing 100038, China; ^2^Clinical Laboratory Medicine, Beijing Shijitan Hospital, Capital Medical University, Beijing 100038, China; ^3^Beijing Key Laboratory of Urinary Cellular Molecular Diagnostics, Beijing 100038, China

## Abstract

**Objective:**

To verify and evaluate the value of CD40 as a noninvasive biomarker of cisplatin-resistant bladder cancer, we studied the expression of CD40 and the correlation between nucleophosmin (NPM1) and CD40 in cisplatin-resistant bladder cancer.

**Methods:**

Three cisplatin-resistant bladder cancer cell lines (T24/0.8DDP, BIU87/0.3DDP, and PUMC-91/0.6DDP) were studied, and lentivirus was used to silence NPM1 expression. The expression of CD40 and NPM1 in three NPM1 silencing bladder cancer cell lines were detected by fluorescence microscopy and Western Blot. The effects and proteomic bioinformatics of NPM1 gene knockout on cisplatin-resistant bladder cancer cells were analyzed by liquid chromatography-mass spectrometry (LC-MS) and gene ontology analysis (GO analysis).

**Results:**

The *NPM1* gene was successfully silenced in three drug-resistant bladder cancer cell lines by lentivirus infection. The knockdown efficiency was 70%. After NPM1 gene knockout, 492 differential proteins were detected by LC-MS, whose fold change was more than 1.5 (*p* < 0.05). A total of 57022 peptides, 54347 unique peptides, and 6686 protein groups were identified in all proteins of the tested cells (FDR < 0.01). Hierarchical clustering and principal component analysis showed that 264 functional proteins were downregulated and 228 functional proteins were upregulated in the gene silencing group compared with those of the negative controls. By GO analysis, the proteins affected by NPM1 cover a large number of proteins with biological functions, which may play an important role in the development of tumor in 492 differential proteins. The CD40 was the most significantly downregulated protein after NPM1 silencing, with a difference of 2.6-fold change in abundance.

**Conclusions:**

There is a positive correlation between CD40 protein and NPM1 protein in drug-resistant bladder cancer. Because NPM1 can reflect the characteristics of bladder cancer, CD40 may be a more sensitive marker for monitoring the prognosis of bladder cancer.

## 1. Introduction

Bladder cancer is one of the most common malignancies of the urinary system and is the most common urinary system tumor in men [1]. At present, the main clinical treatment of bladder cancer is surgery combined with postoperative chemotherapy. However, bladder cancer is prone to drug resistance and recurrence, which makes the cure of bladder cancer more difficult. Early detection and timely intervention can significantly improve the survival rate of patients with bladder cancer. Timely monitoring the progress of drug-resistant bladder cancer is helpful for early targeted treatment of bladder cancer recurrence [2].

At present, the diagnosis and monitoring methods of bladder cancer are mainly urine cytology and cystoscopy. Cystoscopy is an invasive method for detection of bladder cancer, which is easy to cause anxiety and discomfort in patients [3]. Moreover, the sensitivity of cystoscopy to bladder cancer *in situ* (TIS) is low. Urine cytology is a noninvasive diagnostic method, which is less sensitive to small mastoid tumors, satellite lesions, and CIS both in the initial diagnosis and in the monitoring of recurrence. The sensitivity of cystoscopy is low, as low as 28%, and the median is 44% [4]. In addition, the cytological results are also affected by many factors, such as low production of exfoliated cells, urinary tract infection, and calculus, which will affect the clinical interpretation [5].

Urine biomarker detection can greatly improve the sensitivity and specificity of bladder cancer detection, which is a valuable choice. The ideal biomarker of bladder cancer is defined as an objective and noninvasive marker with high sensitivity and specificity [5], but in the existing urine tumor markers, the false-positive results are very common. During hematuria, inflammation, or infection, it is often unable to accurately judge the bladder tumor [3, 6–9].

Nucleophosmin 1 (NPM1) is a kind of protein that mainly locates in the nucleolus and shuttles between the nucleolus and the cytoplasm. Previous studies in our laboratory have shown that NPM1 can reflect specific biological behavior such as recurrence and drug resistance in bladder cancer. The expression of NPM1 in bladder cancer cells increases when the recurrence and drug resistance of invasive bladder cancer cells increase, suggesting that NPM1 may be an important prognostic indicator of bladder cancer cells [10]. However, as a protein only existing in cells, NPM1 has limited sensitivity as early monitoring of bladder cancer. Therefore, it is urgent to find a real-time and effective way to monitor the progress and recurrence of bladder cancer after treatment.

CD40, a transmembrane glycoprotein, is a member of the tumor necrosis factor receptor superfamily. The studies have shown that the CD40 molecule was found on the surface of antigen presenting cells (APC) [11], normal bladder cancer [12], gastric cancer [13], colon cancer [14], and other solid tumors and hematological tumor cells. CD40 molecules are differentially expressed in the process of tumorigenesis and tumor development. CD40 has been regarded as an important biomarker to predict the development in many cancers, such as ovarian cancer [5], hepatocellular carcinoma [6], and other tumors. Ghamade et al. demonstrated that recombinant CD40 ligand therapy had significant antitumor effect on CD40-positive ovarian tumors in mice, and antagonizing CD40 could enhance the effect of cisplatin [8], which means CD40 has a high diagnostic and therapeutic value [7].

CD40 has a variety of oncological activities in tumors, which provides an attractive choice for future clinical applications. As a cell surface secretory molecule, CD40 has a high sensitivity to reflect the biological changes of cells and can predict the biological behavior of tumor cells in the early stage. However, up to now, the differential expression and predictive value of CD40 have not been verified in drug-resistant bladder cancer.

Because NPM1 is closely related to drug-resistant bladder cancer, this study intends to explore the differential expression of CD40 in drug-resistant bladder cancer by mass spectrometry and analyze the correlation between NPM1 and CD40 in drug-resistant bladder cancer, so as to determine whether CD40 is a noninvasive biomarker that can predict the progress of bladder cancer.

## 2. Materials and Methods

### 2.1. Subject and Groups

#### 2.1.1. Drug-Resistant Cell Groups

The cisplatin-resistant bladder cancer cell lines (T24/0.8DDP, BIU87/0.3DDP, and PUMC-91/0.6DDP) were purchased from Beijing Shijitan Hospital Affiliated to Capital Medical University.

#### 2.1.2. NPM1 Differential Expression Cell Groups

NPM1 silencing bladder cancer cell lines (T24/0.8DDP Lv-NPM1, BIU87/0.3DDP Lv-NPM1, and PUMC-91/0.6DDP Lv-NPM1): NPM1-silenced stable infection cell line was constructed by lentivirus infection containing NPM1 shRNA.

NPM1 silencing negative control groups (T24/0.8DDP Lv-NC, BIU87/0.3DDP Lv-NC, and PUMC-91/0.6DDP Lv-NC): the cell lines were constructed by lentivirus infection containing NPM1-negative shRNA.

### 2.2. Materials

0.25% of trypsin (17518012, Gibco Company, USA), RPMI 1640 (AE244464298, Hyclone Company, USA), fetal bovine serum (1861242, Gibco Company, USA), cisplatin (P4394, Sigma Company, USA), polyvinylidene fluoride (PVDF) membranes (ISEQ00010, Sigma Company, USA), mouse anti-nucleophosmin antibody (ab10530, Abcam Company, USA), rabbit anti-CD40 antibody (ab224639, Abcam Company, USA), goat anti-Mouse IgG H&L (HRP) (ab205719, Abcam Company, USA), and NPM1-silencing and NPM1 overexpressing and negative control lentivirus (GenePharma, China) were used.

### 2.3. Methods

#### 2.3.1. Lentivirus Infection and Establishment of Stable Cell Lines

T24/0.8DDP, PUMC-91/0.6DDP, and BIU-87/0.3DDP cells were infected with lentivirus containing NPM1 shRNA (NPM1 interference group) and negative control shRNA (NPM1 negative control group), respectively. The related protein validation and functional experiments were carried out 72 hours after infection. The stable infected cells were screened by single cell cloning. Clones were isolated and screened for stable culture. The isolated clones were amplified and cultured to establish stable cell lines.

#### 2.3.2. Cell Culture

*(1) Drug Resistant Bladder Cancer Cell Groups*. T24/0.8DDP, BIU-87/0.3DDP, and PUMC-91/0.6DDP were cultured in the RPM I1640 medium containing 20% FBS and 0.8 *μ*g/ml DDP, 0.3 *μ*g/ml DDP, or 0.6 *μ*g/ml DDP and kept at 37°C in a 5% CO_2_ saturated humidity incubator, respectively. When cell growth fused into a single layer, the cells were digested and subcultured with 0.25% trypsin.

*(2) NPM1 Silencing Bladder Cancer Cell Groups*. T24/0.8DDP Lv-NPM1, BIU-87/0.3DDP Lv-NPM1, and PUMC-91/0.6DDP Lv-NPM1 were cultured in the RPM I1640 medium containing 20% FBS and kept at 37°C in a 5% CO_2_ saturated humidity incubator, respectively. When cell growth fused into a single layer, the cells were digested and subcultured with 0.25% trypsin.

*(3) The Corresponding Negative Control Cell Groups*. Human bladder cancer cell lines T24/0.8DDP Lv-NC, BIU-87/0.3DDP Lv-NC, and PUMC-91/0.6DDP Lv-NC were cultured in the RPMI 1640 medium containing 15% FBS and kept at 37°C in a 5% CO_2_ saturated humidity incubator, respectively. When cell growth fused into a single layer, the cells were digested and subcultured with 0.25% trypsin.

#### 2.3.3. Western Blot

The total protein was extracted by a RIPA protein lysate. The protein was electrophoretized by SDS-PAGE at 120°V. Then, the protein was transferred to the nitrocellulose filter membranes. The nitrocellulose filter membranes were incubated overnight at 1 : 1,000 anti-NPM1 antibody, anti-CD40 antibody, and anti-*β*-actin antibody. The membranes were incubated at 4°C. The PBST buffer was used to rinse the membranes three times, each 20 minutes, 1 : 1,000 second antibody was added and incubated at room temperature for 90 minutes. The nitrocellulose filter membranes were rinsed by PBST and developed by the DAB kit. All experiments were repeated three times.

#### 2.3.4. Mass Spectrometry

The cell samples were transferred to 1.5 ml microcentrifugal tubes. Then, the dissolving buffer (7 M urea, 2 M thiourea, and 0.1% CHAPS) was added to each sample, and the total protein was extracted by ultrasonic grinding. The supernatant was centrifuged for 30 minutes at 14,000 g for further experiments. Protein concentration was determined by the Bradford method. The appropriate amount of protein (100 *µ*l) was taken for electrophoresis. Gel dyeing and staining were performed until the results were clear. The experiment was repeated three times.

The peptides were separated by a Thermo Scientific EASY-nLC 1,000 system. The peptide mixtures were grouped according to the experimental requirements and labeled with TMT reagents. The mixture of peptides was dissolved in solution A (98% DDH and 2% acetonitrile, pH = 10). Then, the gradient solution B (2% DDH and 98% acetonitrile, pH = 10) was separated in a Durashell-C18 column (4.6 m × 250 m, 5 *µ*m, 100) at a flow rate of 0.7 ml/m. The separation gradient is shown in Table 1. The isolated peptide was detected by a Q-Exactive mass spectrometer (Thermo Scientific). The spray voltage of the ion source was set to 2.1 kV. Full-scanning MS with a resolution of 70,000 was obtained in MS with a resolution of over 350–1,800 *m*/*z*. The spectral resolution of HCD was 17500 fhm. Standardized collision energy was set to 29%.

#### 2.3.5. Proteomic Bioinformatics Analysis

Proteome Discoverer software was used to process the original mass spectrometry data. MS data were searched according to the human genome database and Uniprot website. The parameter settings for MaxQuant search are shown in Table 2.

The biological processes, molecular functions, and cellular components of proteins were analyzed by gene ontology analysis (GO analysis). In addition, the Kyoto Encyclopedia of Genes and Genomes (KEGG) was used to study protein species in order to map the KEGG pathway for systematic functional biology interpretation (http://www.genome.jp/kegg/). Protein-protein interactions are obtained from a search tool that retrieves the Interacting Gene/Protein (String) database (http://string-db.org/), which contains known and predicted physical and functional protein-protein interactions.

### 2.4. Statistical Analysis

All independent experimental data were expressed as mean ± standard deviation (SD). Statistical analysis was carried out by using Graphpad prism 6.0 statistical software Inc. (Lajolla, USA). *p* values were calculated by ANOVA and the Bonferroni test (more than two groups) or *t*-test (two groups). When *p* value<0.05, the results are considered to be statistical.

## 3. Results

### 3.1. Establishment of NPM1 Silencing Stable Cell Lines

**Figure 1:**
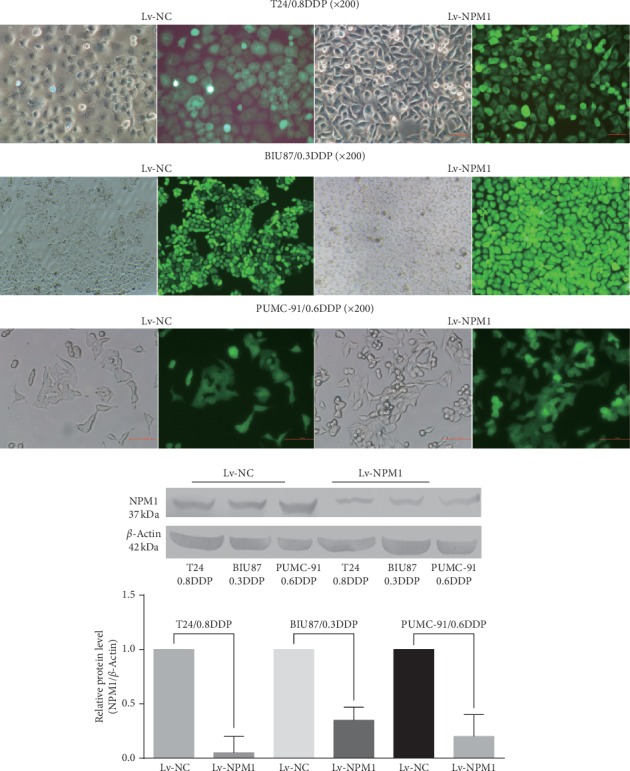
The stable NPM1 silencing cell lines (T24/0.8DDP Lv-NPM1, BIU-87/0.3DDP Lv-NPM1, and PUMC-91/0.6DDP Lv-NPM1) and the corresponding negative control cell lines (T24/0.8DDP Lv-NC, BIU-87/0.3DDP Lv-NC, and PUMC-91/0.6DDP Lv-NC).

We successfully established stable NPM1 silencing drug-resistant bladder cancer cell lines: the fluorescence efficiency of GFP in six kinds of cells (T24/0.8DDP Lv-NPM1, BIU-87/0.3DDP Lv-NPM1, PUMC-91/0.6DDP Lv-NPM1, and the corresponding negative controls) was observed by fluorescence microscopy, which reached more than 80%. Western blot was used to detect NPM1 expression. In the western blot analysis, the level of NPM1 protein in the T24/0.8DDP Lv-NPM1 cell line was 0.05-fold as high as that in T24/0.8DDP Lv-NC (*p* value<0.05). The level of NPM1 protein in the BIU-87/0.3DDP Lv-NPM1 cell line was 0.35-fold as high as that in the corresponding negative control BIU-87/0.3DDP Lv-NC (*p* value<0.05). The level of NPM1 protein in the PUMC-91/0.6DDP Lv-NPM1 cell line was 0.20-fold as high as that in the corresponding negative control PUMC-91/0.6DDP Lv-NC (*p* value<0.05). The experimental results are shown in Figure 1.

### 3.2. Knockdown of NPM1 Gene Resulted in Significant Proteomic Changes in Drug-Resistant Bladder Cancer Cells

**Figure 2:**
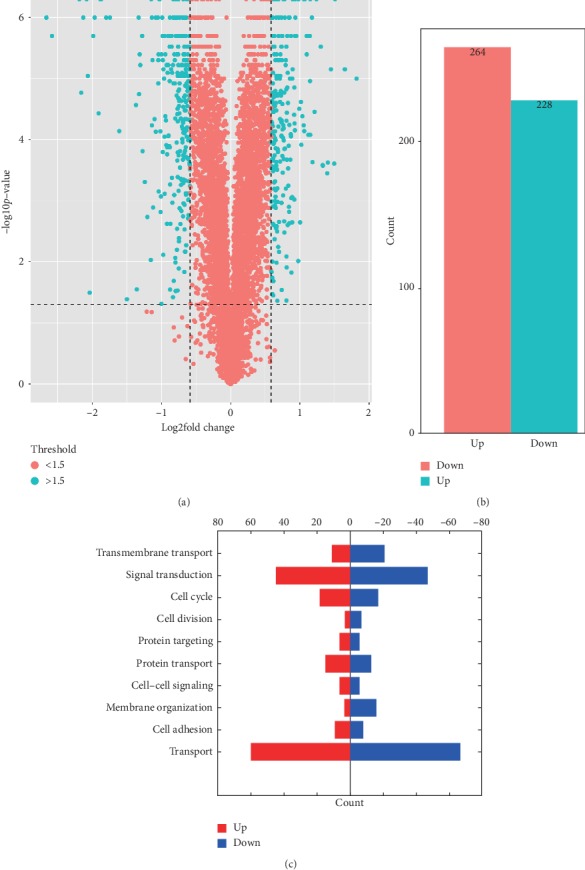
Screening and counting of total differential proteins by mass spectrometry (NPM1 silencing cisplatin-resistant bladder cancer cells and negative control). (a) is the volcanic maps of all proteins present in mass spectrometry. (b) shows the total number of differential screening proteins (fold change >1.5, *p* value<0.05). (c) shows the total number of differential proteins with biological function proved by GO analysis (fold change >1.5, *p* value<0.05).

The effects of NPM1 gene knockout on cisplatin-resistant bladder cancer cells were analyzed by mass spectrometry. A total of 492 differential proteins were identified by principal component analysis. The identified proteins met the following requirements: fold change >1.5 (*p* value<0.05), as shown in Figure 2(a).

The changes of cell proteome were quantitatively recorded by tandem mass tag (TMT) technique. A total of 57022 peptides, 54347 unique peptides, and 6686 protein groups were identified in all proteins of the tested cells (FDR < 0.01). Hierarchical clustering and principal component analysis showed that there were significant protein differences between the gene silencing group and the negative control groups, as shown in Figure 2(b). A total of 264 functional proteins were downregulated and 228 functional proteins were upregulated in the gene silencing group compared with those of the negative controls. We further analyzed the biological functions of 492 differential proteins by GO analysis, as shown in Figure 2(c). These results confirmed that the proteins affected by NPM1 cover a large number of proteins with biological functions. We specifically analyzed the differential protein of the signal transduction protein in drug-resistant bladder cancer, as shown in Table 3.

### 3.3. CD40 Is the Most Significantly Downregulated Protein after NPM1 Silencing in Cisplatin-Resistant Bladder Cancer Cells

After NPM1 silencing, the differential expression of proteins was ranked from high to low in cisplatin-resistant bladder cancer cells and found that downregulation of CD40 expression was the most obvious (fold change: −2.66), as shown in Table 4.

**Figure 3:**
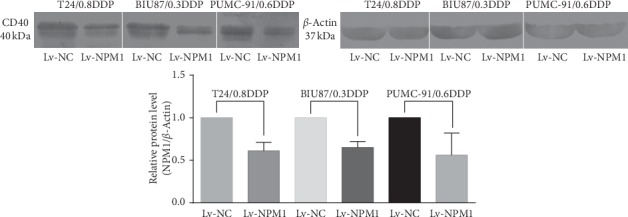
CD40 expression in the NPM1 silencing cisplatin-resistant bladder cell line and the negative control group.

Western blot showed that the expression of CD40 in three NPM1 silencing bladder cancer cell lines was downregulated. In the western blot analysis, the level of CD40 protein in the T24/0.8DDP Lv-NPM1 cell line was 0.61-fold as high as that in T24/0.8DDP Lv-NC (*p* < 0.05). The level of CD40 protein in the BIU-87/0.3DDP Lv-NPM1 cell line was 0.65-fold as high as that in the corresponding negative control BIU-87/0.3DDP Lv-NC (*p* < 0.05). The level of CD40 protein in the PUMC-91/0.6DDP Lv-NPM1 cell line was 0.56-fold as high as that in the corresponding negative control PUMC-91/0.6DDP Lv-NC (*p* < 0.05). This results were consistent with those of mass spectrometry, as shown in Figure 3.

This results were consistent with those of mass spectrometry. This result was confirmed by western blot in three NPM1 silencing bladder cancer cell lines (T24/0.8DDP Lv-NPM1, BIU87/0.3DDP Lv-NPM1, and PUMC-91/0.6DDP Lv-NPM1).

## 4. Discussion

Because bladder cancer is prone to drug resistance and recurrence, it makes it difficult for a reasonable and targeted treatment strategy. Timely screening or monitoring can help doctors prevent and treat the disease as soon as possible.

In order to improve the sensitivity of early diagnosis and alleviate the pain of patients, the researchers are trying to find some noninvasive biomarkers of bladder cancer for clinical practice. Noninvasive biomarkers of bladder cancer have been found to monitor the prognosis of normal bladder cancer [15], such as cytokeratin 18 protein fragment [16]. But there no sensitive and specific biomarker has been found that can early monitor the progress of drug-resistant bladder cancer.

Nucleophosmin (NPM1) is a protein biomarker that can reflect the therapeutic effect of chemotherapy [17], which not only has the function of monitoring the progression in hematological diseases [18, 19], but also has differential expression in some solid tumors such as colon cancer [20] and lung adenocarcinoma [21]. In proteomic analysis of drug-resistant bladder cancer, the expression of NPM1 has been proved to be significantly different [22]. It has also been reported that NPM1 can be used as a valuable prognostic marker of disease [23, 24].

In our previous experiments [10], it has been confirmed that the differential expression of NPM1 can reflect the oncological characteristics of cisplatin-resistant bladder cancer. Downregulation of NPM1 can accelerate the invasion and the growth of cisplatin-resistant bladder cancer. In addition, NPM1 can also reflect the development of cisplatin-resistant bladder cancer. However, as the protein is located in the nucleolus, NPM1 can hardly be monitored as early as to be found as extracellular proteins, which limits its value as a biomarker of tumor in the urine.

CD40, a member of the superfamily of tumor necrosis factor receptors involved in cell differentiation [25], has been proved to be an effective tumor marker in many tumors [26–28], especially in non-B-cell-derived solid tumors, such as nasopharyngeal carcinoma [29], normal bladder cancer [12], cervical cancer [30], and renal cancer [11]. In addition, CD40 has gradually become an effective target for immunotherapy [31].

CD40 can be detected in the exosomes [32]. Some studies have confirmed that CD40 is easy to be monitored early and is related to urinary system diseases such as renal cell carcinoma [9]. However, the value of CD40 in drug-resistant bladder cancer has not yet been evaluated. If we can determine the relationship between CD40 and functional proteins in drug-resistant bladder cancer, we can greatly expand the application of CD40.

To evaluate the value of CD40 as a biomarker in cisplatin-resistant bladder cancer, we established NPM1 silencing stable cell lines by lentivirus infection in three cisplatin-resistant bladder cancer cell lines. According to the research experience, three kinds of drug-resistant bladder cancer can better illustrate the experimental results [33–35]. The results showed that the knockdown efficiency of the *NPM1* gene was about 70%, which suggests that silencing the *NPM1* gene by lentivirus is effective. The most representative T24 cell line in these three kinds of cell lines was further quantitatively analyzed by mass spectrometry. After NPM1 gene silencing, 492 differential proteins were detected by mass spectrometry, whose variation was more than 1.5 times (*p* < 0.05). A total of 57022 peptides, 54347 unique peptides, and 6686 protein groups were identified in all proteins of the tested cells (FDR < 0.01). Hierarchical clustering and principal component analysis showed that 264 functional proteins were downregulated and 228 functional proteins were upregulated in the gene silencing group compared with those of the negative controls. By GO analysis, the proteins affected by NPM1 cover a large number of proteins with biological functions, which may play an important role in the development of tumor in 492 differential proteins. The pathogenesis of oncology often needs to be explained by the changes of the protein in the signal transduction pathway. In MS analysis, the differential expression of NPM1 results in the change of a large number of functional proteins, in which most of them were signal transduction proteins. NF-kappa B signaling pathway can be involved in cell response of cells to external stimuli, such as the effect of cisplatin on drug-resistant bladder cancer. Interestingly, NPM1 has also been proved as an NF-kappaB coactivator for inducting human gene resistance to cell adverse factors [36]. It has been proved that CD40 can regulate the expression of the NF-kappa B signaling pathway through the noncanonical pathway [37, 38].

We further found that CD40 was the most significantly downregulated protein after NPM1 silencing, with a difference of 2.6 times change in abundance, whose expression was positively correlated with NPM1 expression.

**Figure 4:**
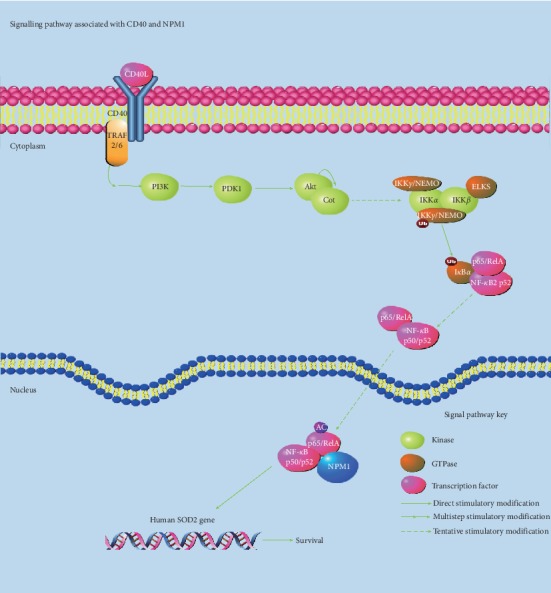
Analysis of signal pathways related to CD40 and NPM1.

The results showed that the variation characteristics of CD40 and NPM1 were consistent. Based on our experimental results and other research studies on NPM1 and CD40, we summarized the related pathways of CD40 and NPM1, as shown in Figure 4. CD40 can be related to the survival, growth, and prognosis of tumors and reflects the changes of the NF-kappa B pathway related to NPM1. In the future, we need further experiments, such as effects of overexpression of CD40 and inhibition of CD40 on the expression of NPM1, to support the claim that NPM1 and CD40 change consistently.

## 5. Conclusion

In view of the downregulation of CD40 expression in cisplatin-resistant bladder cancer cells after NPM1 silencing, CD40 can be related to the survival, growth, and prognosis of tumors and reflects the changes of the NF-kappa B pathway related to NPM1, which indicates CD40 could be used as a biomarker to monitor the progression of cisplatin-resistant bladder cancer.

## Figures and Tables

**Table 1:** Variation of separation gradient of mass spectrometry.

Time (min)	The ratio (%) of gradient solution *B*
0	5
5	8
35	18
62	32
64	95
68	95
72	5

**Table 2:** Parameter settings for MaxQuant search.

Parameters	Value
Enzyme	Trypsin
Static modification	C-carboxyamidomethylation (57.021 Da)
Dynamic modification	M oxidation (15.995 Da);
	N-terminal TMT 9 plex
Species	*Homo sapiens*
Precursor ion mass tolerance	+15 ppm
Fragmentation mass tolerance	+20 MMU
Max missed cleavages	2

**Table 3:** Downregulated proteins with signal transduction function in GO analysis (fold change >1.5, *p* < 0.05).

Id	Accession	Gene name	Description
TNR5_HUMAN	P25942	CD40	Tumor necrosis factor receptor superfamily member 5
NPM_HUMAN	P06748	NPM1	Nucleophosmin
TNR6_HUMAN	P25445	FAS	Tumor necrosis factor receptor superfamily member 6
IF16_HUMAN	Q16666	IFI16	Gamma-interferon-inducible protein 16
BID_HUMAN	P55957	BID	BH3-interacting domain death agonist
E9PCH4_HUMAN	E9PCH4		Uncharacterized protein
DAP1_HUMAN	P51397	DAP	Death-associated protein 1
CNBP1_HUMAN	Q9NSA3	CTNNBIP1	Beta-catenin-interacting protein 1
ASPH_HUMAN	Q12797	ASPH	Aspartyl/asparaginyl beta-hydroxylase
A4_HUMAN	P05067	APP	Amyloid-beta A4 protein
GNAQ_HUMAN	P50148	GNAQ	Guanine nucleotide-binding protein G(q) subunit alpha
K2C8_HUMAN	P05787	KRT8	Keratin, type II cytoskeletal 8
SP110_HUMAN	Q9HB58	SP110	Sp110 nuclear body protein
SDC4_HUMAN	P31431	SDC4	Syndecan-4
CO1A2_HUMAN	P08123	COL1A2	Collagen alpha-2(I) chain
HNRDL_HUMAN	O14979	HNRNPDL	Heterogeneous nuclear ribonucleoprotein D-like
CAB39_HUMAN	Q9Y376	CAB39	Calcium-binding protein 39
ERLN2_HUMAN	O94905	ERLIN2	Erlin-2
CY24 A_HUMAN	P13498	CYBA	Cytochrome b-245 light chain
SLK_HUMAN	Q9H2G2	SLK	STE20-like serine/threonine-protein kinase
CGL_HUMAN	P32929	CTH	Cystathionine gamma-lyase
F5H6H0_HUMAN	F5H6H0	HMGA2	High-mobility group protein HMGI-C
SKI_HUMAN	P12755	SKI	Ski oncogene
BI2L1_HUMAN	Q9UHR4	BAIAP2L1	Brain-specific angiogenesis inhibitor 1-associated protein 2-like protein 1
RHG19_HUMAN	Q14CB8	ARHGAP19	Rho GTPase-activating protein 19
PTEN_HUMAN	P60484	PTEN	Phosphatidylinositol 3,4,5-trisphosphate 3-phosphatase and dual-specificity protein phosphatase PTEN
CCNA2_HUMAN	P20248	CCNA2	Cyclin-A2
PARK7_HUMAN	Q99497	PARK7	Protein/nucleic acid deglycase DJ-1
APOE_HUMAN	P02649	APOE	Apolipoprotein E
IKKA_HUMAN	O15111	CHUK	Inhibitor of nuclear factor kappa-B kinase subunit alpha
RO52_HUMAN	P19474	TRIM21	E3 ubiquitin-protein ligase TRIM21
RWDD1_HUMAN	Q9H446	RWDD1	RWD domain-containing protein 1
AATC_HUMAN	P17174	GOT1	Aspartate aminotransferase, cytoplasmic
ZFY27_HUMAN	Q5T4F4	ZFYVE27	Protrudin
SPTN2_HUMAN	O15020	SPTBN2	Spectrin beta chain, nonerythrocytic 2
SRPRB_HUMAN	Q9Y5M8	SRPRB	Signal recognition particle receptor subunit beta
KBTB7_HUMAN	Q8WVZ9	KBTBD7	Kelch repeat and BTB domain-containing protein 7
DDX54_HUMAN	Q8TDD1	DDX54	ATP-dependent RNA helicase DDX54
A0A0B4J1V8_HUMAN	A0A0B4J1V8	PPAN-P2RY11	HCG2039996
STAT2_HUMAN	P52630	STAT2	Signal transducer and activator of transcription 2
ITPR3_HUMAN	Q14573	ITPR3	Inositol 1,4,5-trisphosphate receptor type 3
TM109_HUMAN	Q9BVC6	TMEM109	Transmembrane protein 109
K7EM85_HUMAN	K7EM85	ARHGAP45	Rho GTPase-activating protein 45
IDE_HUMAN	P14735	IDE	Insulin-degrading enzyme
RADI_HUMAN	P35241	RDX	Radixin
SEM7A_HUMAN	O75326	SEMA7A	Semaphorin-7A
RHG24_HUMAN	Q8N264	ARHGAP24	Rho GTPase-activating protein 24
ERLN1_HUMAN	O75477	ERLIN1	Erlin-1

**Table 4:** The differential expression of proteins in cisplatin-resistant bladder cancer cells after NPM1 silencing.

Differential protein	Ratio
Tumor necrosis factor receptor superfamily member 5 (CD40)	−2.66129
Nucleophosmin (NPM1)	−1.98803

## Data Availability

The data used to support the findings of this study are included within the article.
